# Patterns of cortical thickness alterations in degenerative cervical myelopathy: associations with dexterity and gait dysfunctions

**DOI:** 10.1093/braincomms/fcae279

**Published:** 2024-09-04

**Authors:** Fauziyya Muhammad, Kenneth A Weber, Michael Rohan, Zachary A Smith

**Affiliations:** Department of Neurosurgery, University of Oklahoma Health Sciences Center, Oklahoma City, OK 73104, USA; Systems Neuroscience and Pain Lab, Division of Pain Medicine, Stanford School of Medicine, Palo Alto, CA 94304, USA; Laureate Institute for Brain Research, Tulsa, OK 74136, USA; Department of Neurosurgery, University of Oklahoma Health Sciences Center, Oklahoma City, OK 73104, USA

**Keywords:** degenerative cervical myelopathy, dexterity, mJOA, gait, brain changes

## Abstract

Degenerative cervical myelopathy (DCM) can lead to significant brain structural reorganization. The association between the cortical changes and specific motor symptoms in DCM has yet to be fully elucidated. We investigated the associations between cortical thickness changes with neurological symptoms, such as dexterity and gait abnormalities, in patients with DCM in a case–control study. A 3 Tesla MRI scanner was used to acquire high-resolution T1-weighted structural scans from 30 right-handed patients with DCM and 22 age-matched healthy controls. Pronounced cortical thinning was observed in DCM patients relative to healthy controls, particularly in the bilateral precentral and prefrontal gyri, left pars triangularis, left postcentral gyrus, right transverse temporal and visual cortices (*P* ≤ 0.04). Notably, cortical thickness in these regions showed strong correlations with objective motor deficits (*P* < 0.0001). Specifically, the prefrontal cortex, premotor area and supplementary motor area exhibited significant thickness reductions correlating with diminished dexterity (R^2^ = 0.33, *P* < 0.0007; R^2^ = 0.34, *P* = 0.005, respectively). Similarly, declines in gait function were associated with reduced cortical thickness in the visual motor and frontal eye field cortices (R^2^ = 0.39, *P* = 0.029, R^2^ = 0.33, *P* = 0.04, respectively). Interestingly, only the contralateral precuneus thickness was associated with the overall modified Japanese Orthopaedic Association (mJOA) scores (R^2^ = 0.29, *P* = 0.003). However, the upper extremity subscore of mJOA indicated an association with the visual cortex and the anterior prefrontal (R^2^ = 0.48, *P* = 0.002, R^2^ = 0.33, *P* = 0.0034, respectively). In conclusion, our findings reveal patterns of cortical changes correlating with motor deficits, highlighting the significance of combining objective clinical and brain imaging assessments for understanding motor network dysfunction in DCM.

## Teaser

Muhammad et al. investigated cortical thickness changes in brain regions linked to motor planning in degenerative cervical myelopathy patients. Their novel method revealed patterns correlating cortical changes with motor deficits, highlighting the significance of combining objective clinical and brain imaging assessments for understanding the motor network dysfunction in degenerative cervical myelopathy.

## Graphical Abstract

**Graphical Abstract:**
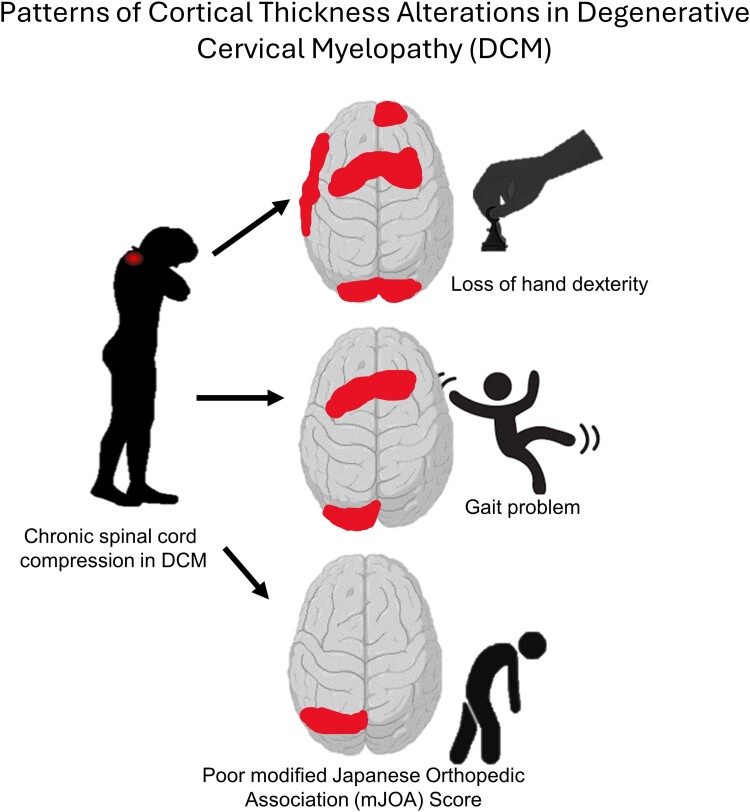

See Nouri, Molliqaj, Schaller and Tessitore (https://doi.org/10.1093/braincomms/fcae322) for a scientific commentary on this article.

## Introduction

Degenerative cervical myelopathy (DCM) results from chronic, progressive spinal cord injury due to degenerative changes of the cervical spine.^1,2^ It is a complex age-related disease characterized by a broad range of symptoms impacting motor and sensory functions. Sensory deficits are far more common and include numbness, altered sensation and pain, which are often the initial symptoms of DCM and may precede motor symptoms. The motor symptoms which include mild weakness in the hands and legs to severe paralysis significantly contribute to DCM morbidity by diminishing a patient's functional capabilities and quality of life.^3^ The neurological changes in DCM are not only localized to the spinal cord, but also to supraspinal abnormalities, suggesting that the latter may emerge as a secondary consequence of the chronic spinal cord damage.^4-8^ These changes in the sensory and motor cortices and other subcortical regions are associated with the severity of DCM.^9,10^

Although sensorimotor cortical alterations in DCM are commonly identified across studies, additional research has documented various other regional alterations in the brain that extend beyond the primary motor and somatosensory pathways.^11^ Woodworth *et al*. showed decreases in precuneus and insula thickness in DCM,^12^ which correlated with the severity of the DCM. Oughourlian *et al*. observed alterations in the brain stem and cerebellum of DCM patients. There have been reports of reduced volumes of subcortical structures and their associations with the Neck Disability Index (NDI) and DCM severity.^12^ The pathophysiology of DCM is suggested by the findings of numerous cortical regional changes.^5,6,13^ However, many of these studies that identified these cortical changes showed inconsistent associations with DCM clinical measures.^12^ The complexity of the brain changes parallels the complexities and intricacies of the presentation of DCM, which can vary significantly between patients with DCM.

A significant limitation of these work is that conventional DCM severity scores were used to analyse brain changes, including the modified Japanese Orthopaedic Association (mJOA) scale and/or the NDI.^7,12^ The mJOA scale categorize DCM patients into mild, moderate or severe based on their symptoms.^12,14^ However, the mJOA scale has significant shortcomings due to the lack of details and granularity that can limit its ability to capture the wide range of presentation in the heterogenous DCM patients. Similarly, the NDI scale focuses on neck pain and its impact on daily activities, but fall short of accurately representing the complexities and varied nature of DCM disabilities^1,15^ in heterogeneous DCM patients. Understanding DCM pathophysiology requires a comprehensive robust assessment that can capture the spectrum and complexities of DCM.^16^ The use of standardized assessment tests that yield quantitative measure on major neurological deficits, such as loss of grip strength, balance and/or gait, as well as loss of hand dexterity has provided more insight into the pathophysiology of DCM.^16,17^ Furthermore, enhancing the accuracy of clinical assessments with robust quantitative measures is key priority in understanding DCM, especially considering the limited predictive power of conventional MR imaging for determining post-treatment recovery.^17-19^ There is a growing emphasis on enhancing clinical assessments and imaging to better understand the complexities of DCM. However, despite this advancement, the relationship between specific DCM deficits evaluated by these improved tools and brain alterations remains largely unexplored.

Understanding the role of brain organizations in DCM is key not only for understanding pathophysiology, it could also potentially offer insights on post-treatment response in DCM. DCM often presents with diverse motor and sensory symptoms, but recognizing the profound impact of motor deficits on DCM patients’ life, we focus on the motor impairment and its relationship with brain changes in DCM. Consequently, delineating the specific cortical regions implicated in each type of motor deficit is critical for understanding the full spectrum of DCM pathophysiology. This study is designed to investigate the hypothesis that distinct patterns of cerebral cortical reorganization are associated with specific motor impairments in DCM. We conducted a focused analysis, examining the association between cortical structural changes and motor deficits quantified through the nine-hole peg test (9HPT) and gait speed assessment. Additionally, we evaluated these cortical alterations in relation to the mJOA score. The results of this study aim to contribute to a more comprehensive framework for interpreting the cortical changes associated with motor deficits in DCM.

## Methods

### Participants: standard protocol approvals, registrations and patient consents

This case–control study was approved by the University of Oklahoma Health Sciences Center (OUHSC) Institutional Review Board (IRB number: 14889), and all experiments were performed in accordance with the Declaration of Helsinki. Clinical and MRI data were collected from 30 patients with different severities of DCM and 22 age-matched HC over the period from 28 May 2021 to 28 July 2023. We utilized convenience sampling to recruit right-handed DCM patients from the OUHSC Neurosurgery Outpatient Clinic during the specified study period. Each patient had an mJOA score of less than 18 and cervical spinal cord stenosis on standard cervical T2W MRI (Supplementary Table 1). Age-matched HC were recruited from the relatives of patients or from volunteer pool of the Laureate Institute for Brain Research (LIBR) site. Written informed consent was obtained from all study participants. DCM patients were included if they had evidence of cervical spinal cord compression on T2-weighted MRI and one more sign of myelopathy that includes (1) hyperreflexia, (2) positive Hoffman sign, (3) loss of hand coordination, (4) gait impairment and (5) motor weakness. All participants were screened by a trained study coordinator to assess the following exclusion criteria: (1) age less than 30 and greater than 70; (2) history of prior spine surgery; (3) three or more comorbid conditions, including, but not limited to, poorly controlled diabetes and hypertension; (4) diagnosis of neurological diseases, such as Parkinson's disease, Alzheimer's, ALS and MS; (5) active systemic rheumatological disease; (6) isolated radiculopathy without myelopathy; (6) history of active peripheral neuropathy; (7) women currently pregnant; (8) history of epilepsy; (9) any medical condition that prevents the completion of any study-related procedure; (10) DCM patients with urgent need for surgical decompression; and (11) participants with asymptomatic spinal cord compression (ASCC) characterized by an mJOA score of 18, cervical canal stenosis and disc herniation or T2 hyperintensity signal on MRI, but without clinical symptoms. Clinical assessments and brain MRI were conducted at the LIBR.

### Clinical assessment

#### All study participants comprising both HC and DCM underwent dexterity test and gait speed test and provided responses to the mJOA scale

The mJOA scoring system evaluates the severity of myelopathy symptoms. It includes a physician-administered questionnaire that focuses on upper extremity motor, upper extremity sensory function, lower extremity motor function and bladder function. Responses are quantified on an 18-point scale, with upper extremity function rated with a maximum of 5 points, lower extremity function with a maximum of 7, sensory function with a maximum of 3 and bladder function also maximum of 3. All study participants were assessed using these examiner-administered mJOA scores, ranging from 0 to 18.^14^

### Dexterity assessment

We implemented the bilateral dexterity test using a nine-hole pegboard (9HT; Jamar 9 Hole Peg Test kit, Performance Health Supply, Inc., Cedarburg, WI) to evaluate hand coordination. This assessment began with the dominant hand, followed by the non-dominant hand. The resulting dexterity scores were calculated as a fully corrected T-scores adjusted for participant demographics, including age and sex, using the NIH Toolbox software.^16^

### Gait speed assessment

Gait speed was measured using the 4-m walk test, a standard tool for assessing locomotion. This metric was recorded in meters per second (m/s), as previously described in Muhammad *et al.* 2023.^16^

#### Brain MRI acquisition

T1-weighted brain images were acquired using the 3T MR 750 GE scanner (GE Healthcare) with an eight-channel head coil (company for the head coil) using the sagittal magnetization prepared rapid gradient echo (MPRAGE) sequence, voxel volume of 1 × 1 × 1 mm^3^ isotropic voxel size and acquisition matrix = 208 × 256 × 256. The total scan duration for T1w images was 6 min 11 s.

#### Image processing and analysis

Cortical parcellation and subcortical segmentation were performed using FreeSurfer 7.2.0 (Surfer.nmr.mgh.harvard) on the T1 images.^20^ The default Freesurfer reconstruction command ‘recon-all’ was run for preprocessing of the MRI data. These preprocessing steps include skull stripping, intensity normalization, motion correction, pial surface and boundary tessellation, cortical parcellation, sub-cortical segmentation, WM segmentation, linear volumetric registration and automated Talairach transformation. The ‘qcache’ option was added to recon all preprocessing to smooth the data. Quality control was performed by visual inspection of the skull stripping and GM and WM boundaries. In addition, the intensity normalization was checked to make sure that the homogenization of signal intensity of the GM and WM is between 100 and 110.

### Statistical analysis

#### Clinical measure analysis

We utilized GraphPad Prism (version 9.5.1) to compare clinical assessment scores between DCM and HC groups.^21^ We performed normality tests with the Shapiro–Wilk test to determine appropriate tests for comparison analyses. The age distributions of DCM and HC along with their dexterity and gait speed measures were compared using Student's *t*-test. The difference in the mJOA scores of the DCM group from a score of 18 was evaluated using one-sample Wilcoxon test. The Fisher's exact test was utilized to assess the differences in sex distribution between HC and DCM groups.

#### Group analysis

We utilized the general linear model (GLM) to evaluate the patterns of interactions and associations between DCM and HC groups. The mris_preproc command was used to concatenate all individual subject data into a single left and right hemisphere datasets using the fsaverage as the target. We performed group analysis by creating contrast matrices and the respective Freesurfer Group descriptor (FSGD) files for GLM analysis. For the group analysis, we utilized the 10 mm full-width half-maximum (FWHMx) smoothing kernels, and the default Different Offset Different Slopes (DODS) method was used to perform group interaction on the Freesurfer ‘mri_glmfit’ command. We performed cortical thickness analysis between DCM and age-matched HC to test the relationship of disease and cortical thickness. We performed cortical thickness interaction with dexterity, gait, complete mJOA scores and mJOA upper and lower extremity motor function sub-scores in DCM patients to determine the slope of the relationships between cortical thickness and severity of DCM.

#### Cluster correction

We utilized Freesurfer ‘mri-glmfit-sim’ command to perform cluster correction with the Monte Carlo simulation technique for multiple comparison corrections. We set the cluster-defining threshold at 1.3 corresponding cluster size threshold to a *P*-value of 0.05 to control the family wise error rate.

#### Region of interest analysis

We performed region-based analysis on cortical thickness of regions that showed significant clusters (region of interest; ROI) with clinical measures. We utilized the ‘aparcstats2table’ command to calculate the measurements of ROI thickness. The cortical parcellation created with the Desikan–Killiany atlas was used for these analyses. Region based statistical analyses were performed using GraphPad prism (version 9.5.1). All analyses were controlled for age where applicable.

#### Multiple linear regression analyses

We performed multiple linear regression analyses for clinical assessment measures, dexterity, gait, mJOA and participants’ age with the region-based measures and cortical thickness of the ROIs identified to yield significance *P* value for each variable and the goodness-of-fit for the model. We performed D'Agostino–Pearson omnibus normality test for normality of the distributed residuals. We performed variance of inflation factors (VIF) to test for multicollinearity for all independent variables to determine the appropriateness of multiple linear regression models.

#### Brain region locator

We utilized the Montreal Neurological Institute (MNI) to Talairach atlas mapping to visualize brain region(s) that showed significant clusters. The respective MNI coordinates were entered into the BioImage Suite Web (https://bioimagesuiteweb.github.io/bisweb-manual/index.html) to identify corresponding Brodmann area(s).

## Results

### Participant characteristics

Our sample included 52 participants: 30 patients with history and MRI evidence of DCM (60% female) and 22 HC (77% female). Statistical analysis using the Fisher's exact test revealed no significant differences in sex distribution between the groups (*P*-value = 0.240). The mean ± SD age of DCM group was 52.08 ± 6.25 years (range 36 to 61 years), and the HC group was 49.09 ± 5.99 (range 36 to 59 years). Participants’ ages were not significantly different between DCM and HC groups (*P* = 0.10). The DCM group presented with poorer mJOA scores (14.00 ± 4.50; range 8 to 17 score) in contrast to the HC group (18.00 ± 0.00; *P* < 0.0001. Additionally, the subscores for both the upper and lower extremities were lower in the DCM group compared to the HC group. The upper extremity mean score was 3.93 ± 0.94 for the DCM group and 5.00 ± 0.00 for the HC group. The lower extremity mean score was 4.93 ± 1.23 for the DCM group and 7.00 ± 0.00 for the HC group. The DCM group exhibited a significant reduction in dexterity (31.60 ± 11.46) compared to the HC group (51.45 ± 9.93), *P* < 0.0001 in the right-hand score and (35.47 ± 12.20) compared to the HC group (48.38 ± 9.61), *P* < 0.0001 in the left-hand score. Additionally, the gait speed for the DCM group was significantly slower (0.70 ± 0.28 m/sec) compared to the HC group (1.04 ± 0.24 m/sec), *P* < 0.0001. Table 1 provides the demographic and clinical characteristics of the participants in both the DCM and HC groups.

**Table 1:** Demographic and clinical information of DCM patients and HC

	HC (n = 22)	DCM (n = 30)	*P*-value
Age (years) mean ± SD	49.09 ± 5.99	52.08 ± 6.25	0.102
Sex (F/M)	17/5	18/12	0.24
mJOA			
Total	18.00 ± 0.00	14.00 ± 4.5	<0.0001
UE	5.00 ± 0.00	3.93 ± 0.94	<0.0001
LE	7.00 ± 0.00	4.93 ± 1.23	<0.0001
Dexterity (T score)			
DH	51.45 ± 9.93	31.60 ± 11.46	<0.0001
NDH	48.38. ± 9.61	35.47 ± 12.20	<0.0001
Gait (m/s)	1.04. ± 0.24	0.70 ± 0.28	0.0002

SD, standard deviation; HC, healthy control; DCM, degenerative cervical myelopathy; F, females; M, males; mJOA, modified Japanese Orthopedic Association score; UE, upper extremity mJOA score; LE, lower extremity mJOA score, DH, right hand score; NDH, left hand score; m/s, meters per seconds.

### Group comparison

#### Cortical thickness changes in DCM

**Figure 1:**
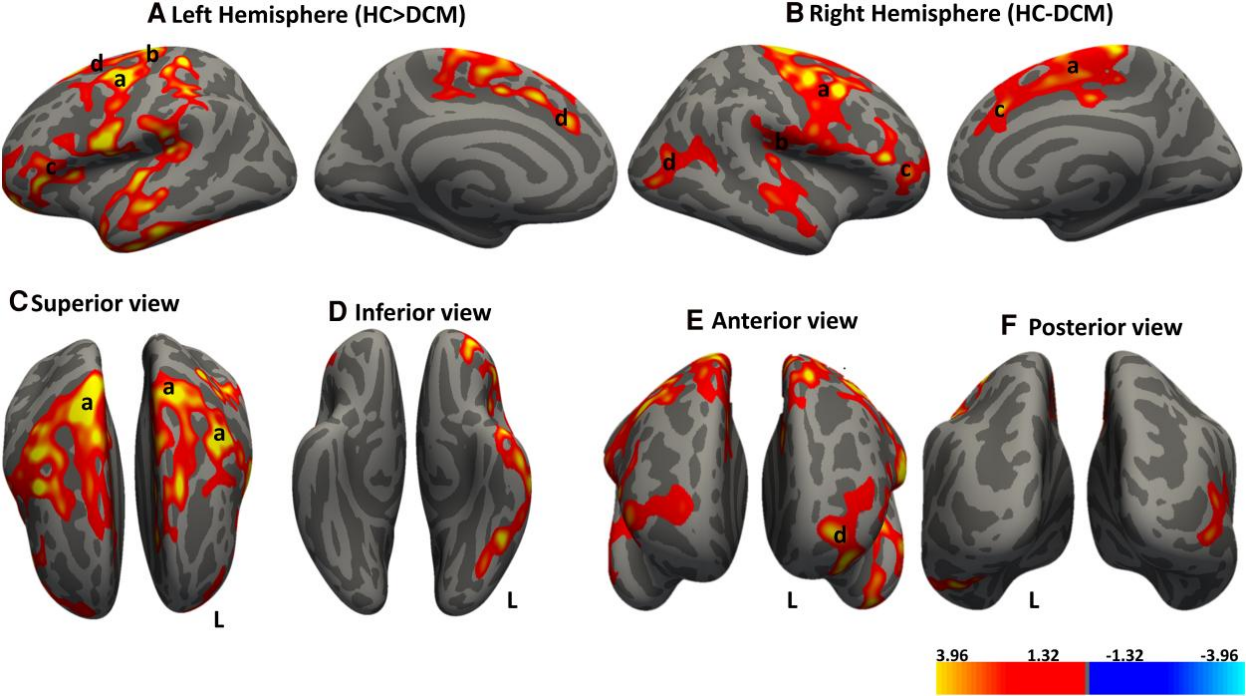
**Cortical thickness reduction in DCM.** Whole brain analysis comparing cortical thicknesses between DCM patients and age-matched healthy controls (HC). Regions demonstrating significant cortical thickness greater in HC compared to DCM (HC > DCM) are shown in red–yellow color. Significant clusters were identified in: (**A**) Left hemisphere: a, premotor cortex and SMA; b, somatosensory cortex; c, pars triangularis of the Broca area; and d, prefrontal cortex. (**B**) Right hemisphere: a, premotor cortex and SMA; b, primary auditory cortex; c, prefrontal cortex; and d, visual association cortex. (**C–F**) Different cortical views. Significant clusters were determined by thresholding at *P* < 0.05. The gradient transitioning from yellow to red denotes regions where cortical thickness is more pronounced in healthy controls (HC, n = 22) compared to DCM (n = 30) patients. General linear model (GLM) ‘mri_glmfit’ and cluster correction with Monte Carlo simulation technique for multiple comparison corrections to control for the family wise error rate (FWER).

Figure 1 shows the results of the whole brain cortical thickness analysis with regions of significant cluster differences between DCM and HC in both left and right cortical hemispheres. We observed that DCM patients exhibited significant decrease in cortical thickness in bilateral precentral gyri, bilateral rostral middle frontal gyrus (RMFG; prefrontal cortices), left postcentral gyrus (somatosensory cortex), left par triangularis (Broca area), right transverse temporal cortex, lateral occipital gyrus and right hemisphere. Table 2 provides a summary of the respective cluster sizes (mm) and MNI coordinates of the cortical regions that are significantly different in thickness between patients with DCM and HC.

**Table 2:** Summary of regions showing significant differences in cortical thickness between DCM and HC

Hemisphere	Cortical region	Cluster size (mm^2^)	*P*-value	Peak x y z	MNI to Talirach
Left	Precentral	7617.78	0.0002	−40.6 −7.3 50.8	Premotor and SMA
	Postcentral	6644.83	0.0002	−35.6 −35.1 60.5	Primary sensory area
	Pars triangularis	1314.19	0.0195	−45.2 31.6 −2.5	Broca area
	Rostral middle frontal	1188.37	0.0365	−29.6 51.3 −11.2	Prefrontal cortex
Right	Precentral	10 777.11	0.0002	10.2 −25.8 71.9	Premotor and SMA
	Transverse Temporal	1349.96	0.0086	39.4 −27.8 11.3	Primary auditory
	Lateral occipital	1076.65	0.0392	46.0 −79.6 1.5	Visual association cortex
	Rostral middle frontal	1054.81	0.0422	40.2 41.2 1.9	Prefrontal cortex

DCM, degenerative cervical myelopathy; HC, healthy controls; SMA, supplementary motor area.

#### Cortical thickness changes and associations clinical assessment measures

Multiple linear regression analysis revealed an association between cortical thickness in specific ROIs as outlined in Table 2 and the clinical assessment measures. Dexterity measures emerged as the only significant clinical assessment predictors of cortical thickness in the bilateral prefrontal cortex, bilateral premotor and SMA and the left Broca area, as indicated in Table 3. Additionally, our results showed no significant association between the mJOA and gait scores and the thickness of these ROIs. Furthermore, we observed that cortical thinning in the ROIs increased with advancing age in the study participants.

**Table 3:** Multiple linear regression of cortical measures with clinical scores

Measure	Hemisphere	Cortical region	Dexterity *P* value	Gait *P* value	mJOA *P*-value	Age *P* value	Overall model R^2^	Overall model *P*-value
Cortical thickness (mm)	Left	Premotor and SMA	0.005	0.772	0.232	0.001	0.34	<0.0001
	Left	Primary sensory	0.1066	0.9306	0.8241	0.1132	0.1	<0.0001
	Left	Broca area	0.0277	0.9028	0.9645	0.0002	0.2764	<0.0001
	Left	Prefrontal	0.0007	0.5546	0.4341	<0.0001	0.3305	<0.0001
	Right	Premotor and SMA	0.0222	0.8466	0.0884	0.0007	0.3658	<0.0001
	Right	Primary auditory	0.4621	0.7556	0.1298	0.0137	0.2062	<0.0001
	Right	Visual association	0.8456	0.7066	0.2525	0.5439	0.0387	<0.0001
	Right	Prefrontal	0.0023	0.5005	0.9877	0.0004	0.2864	<0.0001

SMA, supplementary motor area; mJOA, modified Japanese Orthopedic Association score.

mJOA, modified Japanese Orthopedic Association score.

#### DCM severity and association with cortical thickness changes

**Figure 2:**
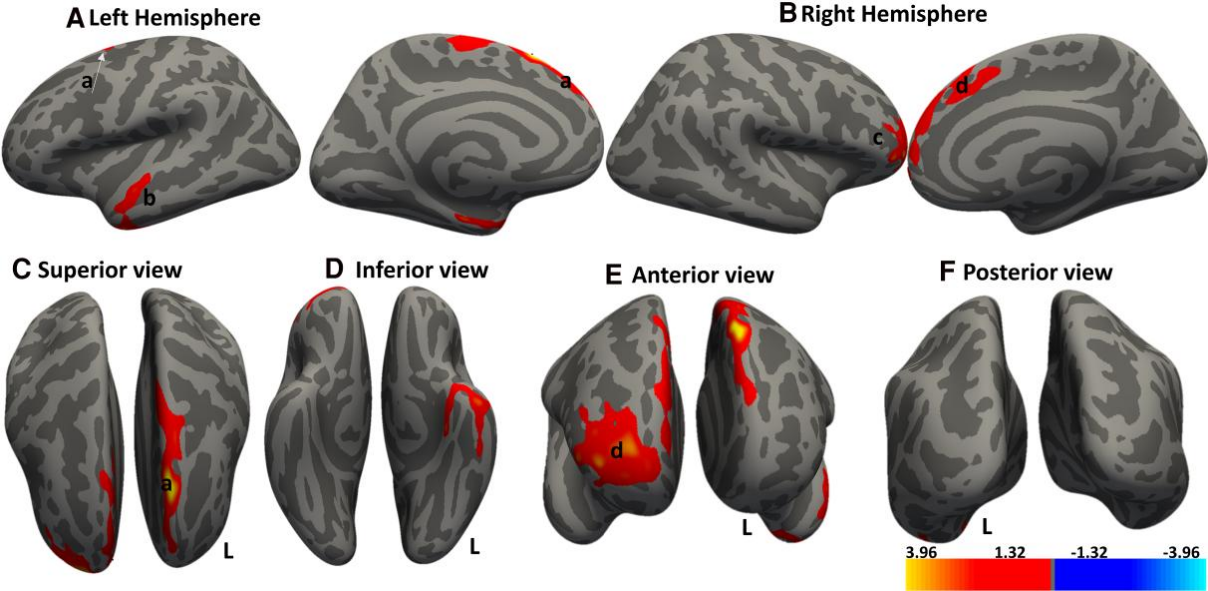
**Cortical regions show significant positive associations between cortical thickness and dominant hand dexterity score.** Whole brain analysis determines the association between cortical thickness and dexterity score. Red–yellow denotes increasing cortical thickness with increasing dexterity score. Regions that show significant clusters in the (**A**) left hemisphere are *a*, superior frontal region and b, inferior temporal region, while those for the (**B**) right hemisphere are *c*, prefrontal cortex and *b*, superior frontal. (**C–F**) Different cortical views. Significant clusters were determined by thresholding based on the statistical significance of *P* < 0.05. The gradient transitioning from yellow to red denotes a positive correlation between cortical thickness and covariate (dominant hand dexterity score) in DCM patients (n = 30). General linear model (GLM) ‘mri_glmfit’ and the dexterity scores of the dominant hand used as a covariate within the GLM framework. cluster correction with Monte Carlo simulation technique for multiple comparison corrections to control for the family wise error rate (FWER).

**Figure 3:**
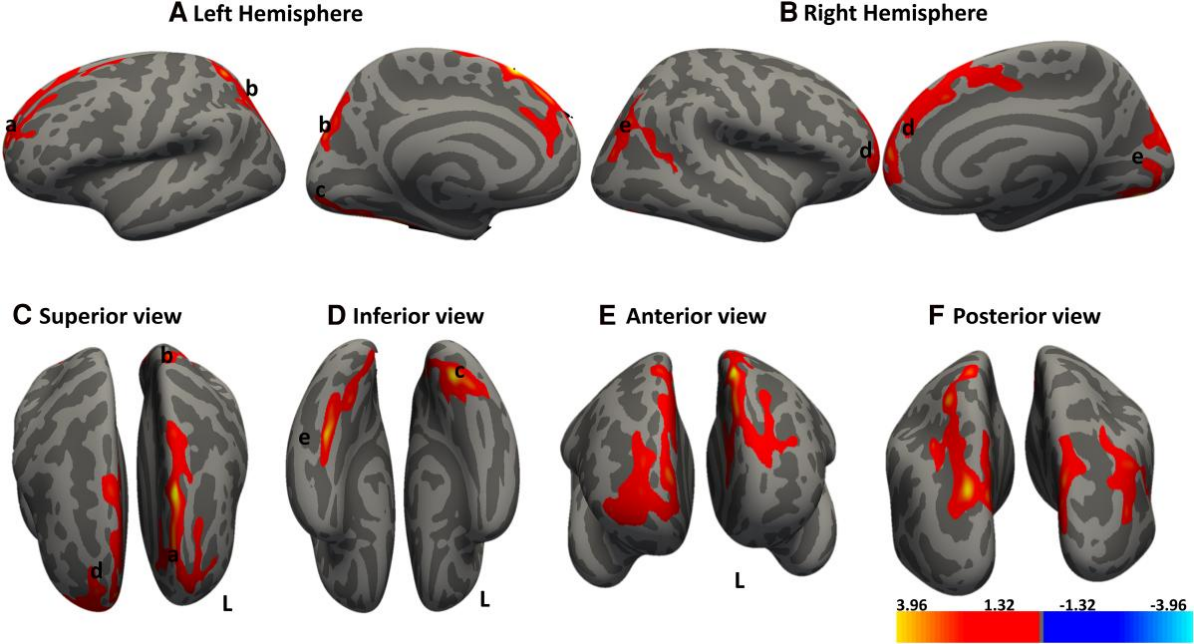
**Cortical regions show significant positive associations between cortical thickness and non-dominant dexterity score.** Whole brain analysis determines the association between cortical thickness and dexterity score. Red–yellow denotes increasing cortical thickness with increasing dexterity score. Regions that show significant clusters in the (**A**) left hemisphere are a superior frontal region, b, superior parietal and c, fusiform cortex, while those in the (**B**) right hemisphere are d anterior prefrontal and e, secondary visual. (**C–F**) Different cortical views. Significant clusters were determined by thresholding based on the statistical significance of *P* < 0.05. The gradient transitioning from yellow to red denotes a positive correlation between cortical thickness and covariate (non-dominant hand dexterity score) in DCM patients (n = 30). General linear model (GLM) ‘mri_glmfit’ and the dexterity scores of the dominant hand used as a covariate within the GLM framework. Cluster correction with Monte Carlo simulation technique for multiple comparison corrections to control for the family wise error rate (FWER).

**Figure 4:**
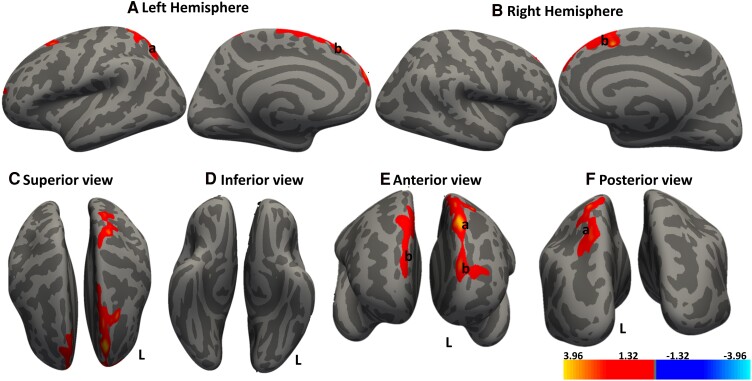
**Cortical regions show significant positive associations between cortical thickness and gait score.** Whole brain analysis determining the association between cortical thickness and gait score. Red–yellow denotes increasing cortical thickness with increasing gait score. Regions that show significant clusters in the (**A**) left hemisphere are a, superior parietal and b, superior frontal area, while that for the (**B**) right hemisphere is b, superior frontal area. (**C–F**) Different cortical views. Significant clusters were determined by thresholding based on statistical significance of *P* < 0.05. The gradient transitioning from yellow to red denotes a positive correlation between cortical thickness and covariate (gait speed score) in DCM patients (n = 30). General linear model (GLM) ‘mri_glmfit’ and the dexterity scores of the dominant hand used as a covariate within the GLM framework. Cluster correction with Monte Carlo simulation technique for multiple comparison corrections to control for the family-wise error rate (FWER).

**Figure 5:**
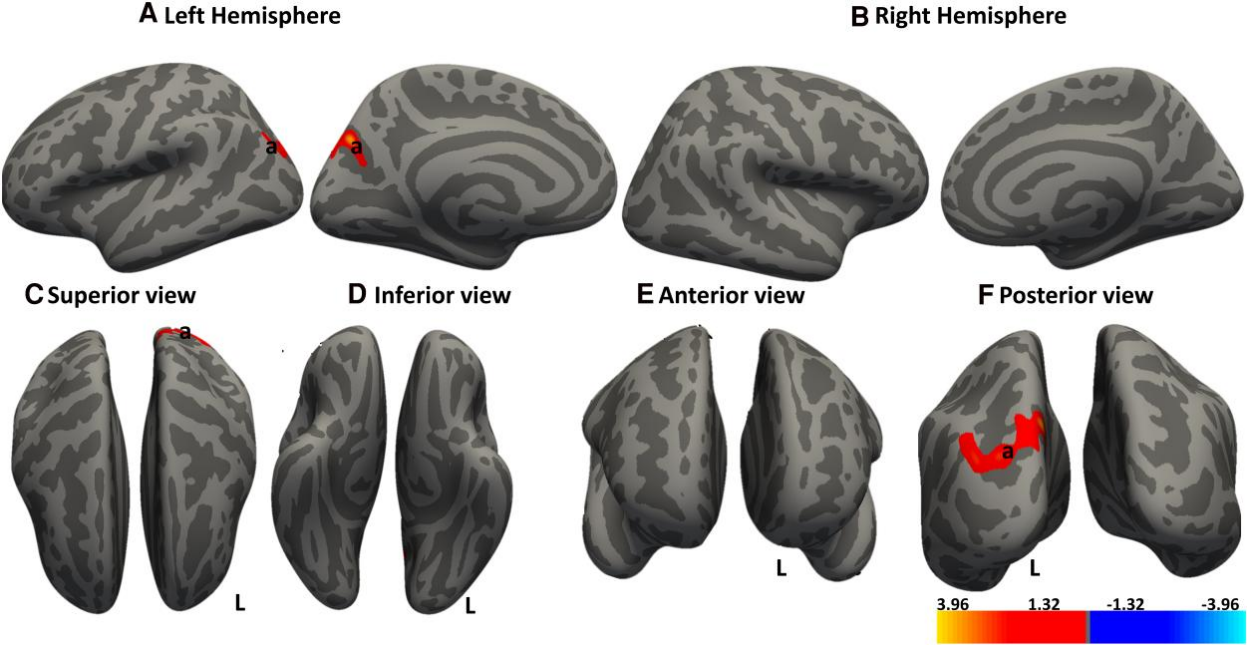
**Cortical regions show significant positive associations between cortical thickness and mJOA score.** Whole brain analysis determines the association between cortical thickness and mJOA score. Red–yellow denotes increasing cortical thickness with increasing mJOA score. Region that shows significant cluster in the (**A**) left hemisphere is a, precuneus area. (**B**) Right hemisphere. (**C–F**) Different cortical views. Significant clusters were determined by thresholding based on the statistical significance of *P* < 0.05. The gradient transitioning from yellow to red denotes positive correlation between cortical thickness and covariate (composite mJOA score) in DCM patients (n = 30). General linear model (GLM) ‘mri_glmfit’ and the dexterity scores of the dominant hand used as a covariate within the GLM framework. Cluster correction with Monte Carlo simulation technique for multiple comparison corrections to control for the family-wise error rate (FWER).

**Figure 6:**
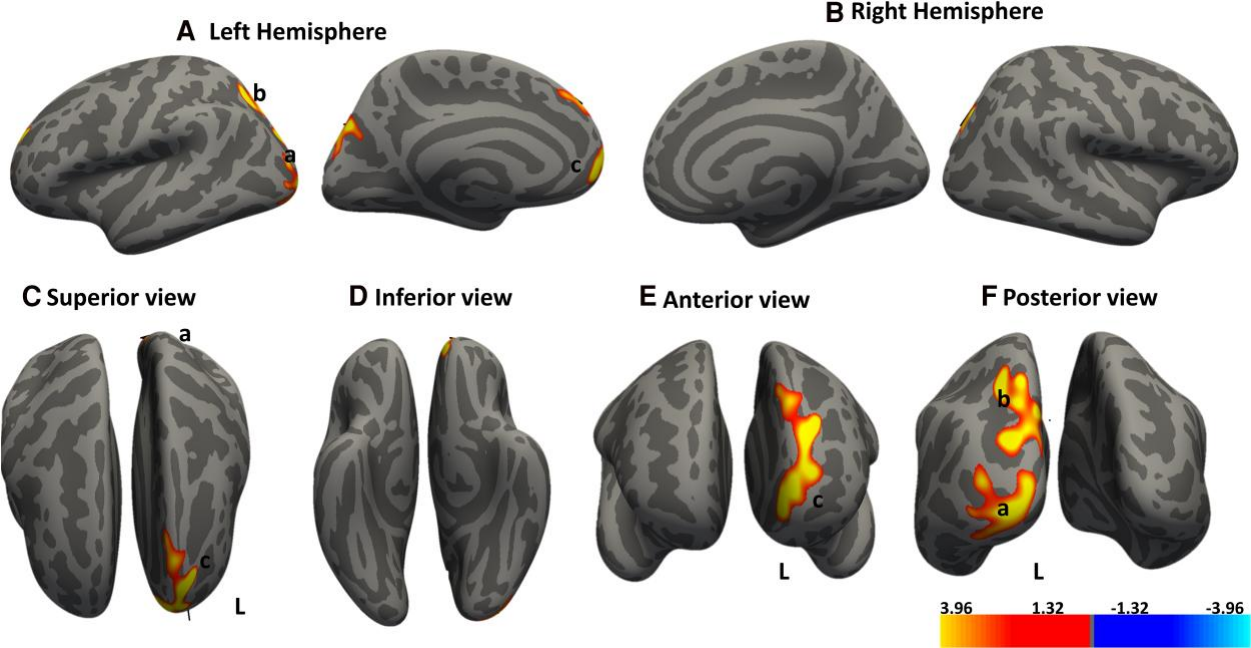
**Cortical regions showing significant positive associations between cortical thickness and mJOA upper extremity sub-score.** Whole brain analysis determines the association between cortical thickness and mJOA sub-score. Red–yellow denotes increasing cortical thickness with increasing dexterity score. Regions that show significant cluster in the (**A**) left hemisphere are a, secondary visual, b, superior parietal and c, anterior prefrontal. (**B**) No cortical area identified on the right hemisphere. (**C–F**) Different cortical views. Significant clusters were determined by thresholding based on statistical significance of *P* < 0.05. The gradient transitioning from yellow to red denotes a positive correlation between cortical thickness and covariate (mJOA upper extremity sub-score) in DCM patients (n = 30). General linear model (GLM) ‘mri_glmfit’ and the dexterity scores of the dominant hand used as a covariate within the GLM framework. Cluster correction with Monte Carlo simulation technique for multiple comparison corrections to control for the family-wise error rate (FWER).

In the DCM cohort, we performed vertex-based correlation analyses to explore the relationship between cortical thickness and severity of DCM symptom. Our results identified specific cortical regions where changes in thickness directly correlated with the clinical assessment measures, as shown in Figs. 2–6.

We observed distinct clusters in the brain where a decreased cortical thickness is positively correlated with the decreasing clinical measure listed in Table 4. Notably, we found seven clusters across both hemispheres that exhibited a significant positive correlation with dexterity scores of the dominant hand. These clusters were located in the frontal eye field region, prefrontal cortex and inferior temporal regions, as shown in Fig. 2. Interestingly, with the non-dominant hand, we observed significant correlations in the prefrontal cortex, bilateral visual association and secondary visual areas, Fig. 3. These patterns suggest that as dexterity decreases, there is a corresponding reduction in cortical thickness within these regions.

**Table 4:** Summary of significant clusters from vertex-based analysis with clinical assessment regressors

Clinical measure		Hemisphere	Cortical region	Cluster size (mm^2^)	*P* value	Peak x y z	Brain location
Dexterity	Right Hand	Left	Superior frontal	1634.46	0.003	−9.2 33.3 52.1	Frontal eye field
			Inferior temporal	1584.78	0.005	−34.5 −0.3 46.4	Inferior temporal
		Right	Rostral middle frontal	2028.65	0.004	39.9 −16.6 57.2	Anterior prefrontal
			Superior frontal	1233.52	0.03	10.9 53.8 8.8	Frontal eye Field
	Left Hand	Left	Superior frontal	2572.1	0.000	−7.2 26.6 56.4	Frontal eye Field
			Superior parietal	2554.94	0.000	−22.7 −82.6 28.6	Visual association
			Fusiform	1215.48	0.030	−36.3 −50.1 −18.8	Visual motor
		Right	Superior frontal	3370.64	0.000	10.2 58.8 11.1	Anterior prefrontal
			Lingual	1534.31	0.007	20.6 −81.5 −11.7	Secondary visual
			Inferior parietal	1438.14	0.013	40.8 −78.2 16.3	Visual association
			Superior parietal	1163.64	0.045	16.1 −84.0 37.3	Visual association
Gait Speed		Left	Superior frontal	2112.27	0.0002	−8.8 41.4 44.4	Frontal eye Field
			Superior parietal	1242.08	0.0002	−20.3 −47.9 58.8	Visual motor
		Right	Superior frontal	1193.1	0.0002	8.2 24.6 39.5	Frontal eye Field
mJOA	Overall	Left	Precuneus	1256.62	0.0028	−16.2 −71.1 34.3	Visual motor
		Right	No region identified				
	Upper Extremity Score	Left	Superior parietal	1742.86	0.0018	−22.2 −81.7 17.6	Visual association
			Rostral middle frontal	1643.32	0.0034	−20.3 49.6 26.8	Anterior prefrontal
			Lateral occipital	1500.9	0.009	−22.0 −98.5 −1.8	Secondary visual
		Right	No region identified				
	Lower extremity score	No region identified					

Interestingly, the gait scores displayed significant positive correlations with cortical clusters in the bilateral frontal eye field and the left visual cortex, Fig. 4. This implies that cortical thickness of the visual cortex is associated with gait performance.

Additionally, the correlation analysis with mJOA scores revealed a significant positive correlation only in a cluster located in the left precuneus region, a segment of the visual cortex, Fig. 5. On further investigation of the mJOA sub scores, it was found that the upper extremity score showed an association with regions in the visual association and secondary visual areas, as well as the prefrontal cortex, Fig. 6. Contrarily, no cortical area association was detected with the lower extremity sub score.

## Discussion

This study is distinctive in its exploration of the intricate patterns of brain changes in DCM patients, focusing on how cortical thinning is associated with various motor deficits. Specifically, we focus on the roles of the different cortical regions that undergo changes with increasing severity of DCM dysfunctions. The findings from this study mark a significant advancement from existing studies on brain changes in DCM, providing a deeper understanding of how these cortical changes correlate with specific motor impairments.^6,10,15^ By contrasting these novel findings of brain changes with correlation with symptom assessment with different scales (mJOA, dexterity measure and gait speed), our study sheds light on the complex relationship between motor dysfunction and cortical changes in DCM. Correlating cortical changes with motor deficits highlights the significance of combining objective clinical and brain imaging assessments for understanding complex motor network dysfunction in DCM.

Our results reveal cortical thickness reduction across several key regions, including the premotor, supplementary motor (SMA), prefrontal and somatosensory regions and visual and auditory cortices in patients with DCM. These regions are crucial for motor control.^22^ Reduced cortical thickness in the prefrontal and premotor regions indicates dysfunction of the brain areas involved in motor planning and coordination of intricate and sequential movements. Not surprisingly, these patients also have significant impairments in gait and hand function, which require precise and intricate movements and coordination. Furthermore, the SMA plays a role in the preservation of posture during motor movements involve during gait.^23^ The alterations in the thickness of the motor cortex are consistent with results from other studies that have described the structural and functional changes in the motor cortex due to chronic spinal cord compression.^7,13^ Our results not only showed cortical thickness reduction in these motor regions, but also elaborated on the presence of multiple patterns of cortical thickness changes that have not been previously discussed. Furthermore, the identification of changes in these regions integral to motor processing underscores the significant role that these areas play in DCM pathology. The reduction in cortical thickness in these regions may reflect brain atrophy, providing a possible explanation for the persistent motor deficits observed in DCM patients, even after surgical intervention. Our results align with those of other studies that have demonstrated cortical changes and adaptation in DCM.^5,9,24,25^ This consistency across studies reinforces our understanding of the impact of DCM on the brain, particularly in the context of motor function impairment and potential cortical resilience in specific areas.

To gain insight into the severity of DCM symptoms and how it impacts cortical reorganization, we focused on the objective quantitative assessment of upper and lower extremity dysfunction that examined gait, hand coordination and weakness.^16^ DCM patients performed worse than HC in different motor assessment tests, including dexterity and gait tests.^16,17,19^ We observed that cortical thickness in the prefrontal, premotor and SMA regions was associated with dexterity. We noted a reduction in the thickness of the visual cortex with gait and dexterity. These cortical changes further highlight visuomotor integration during object-directed motor functions.^26^ Existing research has revealed functional alterations in DCM's visual cortex.^10,27^ It is noteworthy to mention that ipsilateral clusters were found in the secondary motor areas in this study based on right-sided motor function measurements. These findings are consistent with that earlier research showing that the ipsilateral hemisphere is involved in the planning and execution of specific movements.^28^ These results collectively offer insight into the neuroanatomical consequences of DCM that aligns with the spectrum of motor and sensory impairments. While our focus in dexterity captured fine motor skills,^16^ future research should incorporate border assessment that integrates strength, coordination and prehension (GRASSP test).^19^ This comprehensive technique will provide more extensive understanding of the complex relationships between cortical changes and hand function in DCM.

Using clinical assessment group regression and cortical thickness interaction, we hypothesized that distinct motor deficits could be used to predict distinct patterns of cortical thickness change. Our regression model results show that only dexterity measures can predict cortical thickness in many regions involved in motor processing. The prefrontal region's function in fine motor planning and coordination as well as hand coordination as measured by the dexterity score appears to be supported by these findings.^29^ This conclusion is consistent and further emphasizes the involvement of the prefrontal cortex in high-precision activities, such as hand coordination and dexterity.^30^ The findings that gait performance associated with the cortical thickness changes in the visual cortices indicate a possible interplay between visual processing and motor function in DCM.

Our study suggests that while the mJOA score is useful for assessing general DCM severity, it does not correlate with motor cortical changes but with a region of the visual cortex. This aligns with other studies that elaborated the limitations of mJOA scores in predicting cortical thickness changes.^12^ The mJOA scale provides a general assessment of the severity of DCM by calculating scores from the lower and upper extremities, pain and bladder function.^14^By analyzing the mJOA sub-score, we found that the upper extremity sub-score was correlated with additional visual areas and the prefrontal cortex not seen with the lower extremity sub scores. This contrasts with the findings for the lower extremity mJOA, suggesting a potential limitation in the ability of the composite mJOA to accurately assess lower extremity deficits in DCM. Therefore, our findings imply that the heterogeneity of DCM presentation may be better captured by analysing the mJOA sub scores than relying on the composite score.

Furthermore, prior studies primarily utilized patient-reported clinical assessment scales, including the mJOA, NDI or Nurick grade, which are focused on assessing the overall clinical severity of DCM.^14,31^ The reliance on assessment scales that focus on subjective aggregate of DCM severity may overshadow the nuanced understanding of specific DCM impairments and their impact in cortical changes. Our study advocates for the utilization of more objective assessment measures, such as the dexterity and gait assessments, as well as mJOA sub scores, which describe distinct motor deficits in DCM more accurately than composite assessment tests. This approach emphasizes the importance of examining specific impairments in DCM and their neuroanatomical correlates. Our research findings align with a global research priority for DCM.^32^

This study has some limitations that must be noted. First, only changes in cortical thickness were included in this study. DCM has been shown to involve subcortical structural abnormalities in addition to cortical changes in the brain.^4^ It is critical to assess subcortical alterations and the effects of any modifications on the motor symptoms of DCM. Functional changes in the brain stem, basal ganglia nuclei and thalamus have been described previously.^25,33^ Second, sensory symptoms, including pain, which are highly prevalent and are important DCM symptoms, were not examined in this study.^12^ Expanding this study with a larger sample size is necessary to corroborate these results and enable a broader interpretation and clinical application of the findings. Third, this study only included right-handed participants; therefore, it is critical to determine whether the identified cortical clusters are affected by the laterality of DCM or the handedness of the patients. Further research is necessary to fully understand the intriguing findings of hemispherical cluster responses based on hand dominance. Fourth, the symptom duration of the patients included in the study is not known. The relationship of the symptom duration and the brain changes could provide important insight into the DCM phenotype and supraspinal changes. Lastly, although it is unknown whether there is an increased sex propensity to DCM, certain studies have detailed sex variations in the cortical response in DCM.^10^ However, sex differences were not investigated in this study.

Further research is required to determine which patient responses stem from long-term structural alterations. Examining post-surgery brain alterations in DCM and their connections to particular clinical impairments would also be worthwhile. Longitudinal studies may provide insight into the evolution of structural and functional changes over time. Participants with asymptomatic spinal cord compression (ASCC) were excluded from this study. Expanding the study to include patients with ASCC, diverse neurological profiles and different hand dominances would provide a more holistic understanding of DCM. It is crucial to determine whether HC, ASCC and DCM have different cortical thicknesses and how they change over time.

In conclusion, our findings serve as a pivotal step towards understanding the distinct cortical changes that underpin specific motor deficits in DCM. The mechanism, by which a particular pattern of cortical alterations in the brain associates a particular DCM motor deficit, has not received much attention until this study. This study describes the relationship between cortical alterations and motor impairments in DCM by using a standardized, repeatable, quantitative and reliable scale that indicates the type and extent of motor dysfunction. This contrasts with many other studies that rely on subjective scales to assess the global dysfunction of DCM.^12^ Our research may contribute to a better understanding of the brain alterations underlying the heterogeneity in DCM and the reasons for the variability in DCM recovery. The results of structural alterations showed brain cortical atrophy in DCM, which may provide a clue as to why most DCM patients do not experience complete symptom improvement after surgical decompression.

## Supplementary Material

fcae279_Supplementary_Data

## Data Availability

The data are available upon reasonable request to the senior author.
