# Supplementary material for: Patterns of cortical thickness alterations in degenerative cervical myelopathy: associations with dexterity and gait dysfunctions
Source: Brain Commun. 2024 Sep 4;6(5):fcae279. doi: 10.1093/braincomms/fcae279 (PMC11448325; doi:10.1093/braincomms/fcae279)
Supplement: fcae279_Supplementary_Data [file fcae279_supplementary_data.docx]

**Supplementary Table 1**. The spinal cord cross sectional area (CSA) assesses the degree of cervical spinal cord compression, with the maximum compression level MCL representing the most compressed vertebral level observed. The CSA values are methodically derived by averaging the measurements from the initial to the final slice of the affected vertebral level. The average CSA for the DCM group was found to be 59.23 mm² (SD 9.35mm²), compared to the HC group, 74.44 mm² (SD 7.78 mm²). MCL, maximum compression level, CSA cross sectional area, DCM, degenerative cervical myelopathy, HC, healthy control

| **Supplementary Table 1** | | |
| --- | --- | --- |
| **DCM Participants** | **MCL** | **CSA (**mm^2)^ |
| 1 | C4 | 61.25 |
| 2 | C3 | 55.90 |
| 3 | C4 | 66.08 |
| 4 | C6 | 60.94 |
| 5 | C6 | 54.45 |
| 6 | C4 | 26.08 |
| 7 | C6 | 47.77 |
| 8 | C5/C6 | 52.96 |
| 9 | C5 | 65.30 |
| 10 | C5/C6 | 47.36 |
| 11 | C7 | 50.03 |
| 12 | C3 | 44.62 |
| 13 | C5/C6 | 53.22 |
| 14 | C6 | 44.44 |
| 15 | C5/C6 | 52.92 |
| 16 | C5 | 43.55 |
| 17 | C6 | 64.70 |
| 18 | C6 | 32.68 |
| 19 | C5/C6 | 54.51 |
| 20 | C6 | 55.81 |
| 21 | C5 | 48.94 |
| 22 | C5 | 48.42 |
| 23 | C5/C6 | 63.65 |
| 24 | C6 | 42.25 |
| 25 | C6 | 49.36 |
| 26 | C6 | 41.61 |
| 27 | C5/C6 | 42.42 |
| 28 | C5 | 55.88 |
| 29 | C6 | 50.71 |
| 30 | C5 | 52.79 |

MCL, maximum compression level, CSA cross sectional area,

DCM, degenerative cervical myelopathy, HC, healthy control
